# CENP-H as a new prognostic biomarker for tumors: a real-world literature review

**DOI:** 10.3389/fonc.2025.1521988

**Published:** 2025-02-25

**Authors:** Qiao Li, Yang Tang, Jie-Bin Zuo, Hao Han, Guang-Xu Tu, Cheng Chen

**Affiliations:** Department of Thoracic Surgery, Affiliated Hospital of Zunyi Medical University, Zunyi, China

**Keywords:** CENP-H, cancer, poor prognosis, biomarker, lncRNA

## Abstract

Centromere protein H (CENP-H) is an important component of a functional centromere. Studies have demonstrated that CENP-H is overexpressed in renal cell, gastric, hypopharyngeal squamous cell, nasopharyngeal, endometrial, lung, cervical, esophageal, liver, colorectal, oral squamous cell, breast, and tongue carcinomas. CENP-H overexpression is positively correlated with a poor prognosis, pathological stage, T stage, and lymph node metastasis in patients with the above carcinomas. CENP-H can promote cancer growth and metastasis through PI3K/AKT, survivin, and mitochondrial apoptosis signaling mechanisms, and it can be regulated by long non-coding ribonucleic acid (lncRNA) plasmacytoma variant translocation 1 (PVT1)/miR-612, Sp1, or Sp3. This review aims to summarize the expression of CENP-H, the relationship between CENP-H expression and prognostic features, growth and metastasis of cancer in patients, as well as the mechanism of CENP-H in cancer. It also proposes a new candidate molecule for treating patients with cancer.

## 1 Background

Cancer biomarkers are specific molecules or cells detected in tissue and blood samples when cancer develops inside the body. The levels of these biomarkers vary in a patient’s body during the early stages of cancer growth. They can, therefore, be used to aid early cancer screening and diagnosis. Detecting these markers allows timely identification of potential cancer risk in a patient. During cancer treatment, analyzing the level of these biomarkers can help monitor the disease progression and the therapeutic effect, as well as evaluate the prognosis of patients with cancer and formulate more appropriate treatment plans (1–4). For instance, Chen et al., observed that gamma-glutamyl transferase 5 (GGT5) was highly expressed in gastric cancer (GC) tissues, which was associated with poor prognosis and clinical staging of patients with GC. Downregulating GGT5 expression inhibited the proliferation and migration of GC cells by targeting the PI3K/AKT pathway (1). Such markers can be used to aid cancer diagnosis and assess patient outcomes.

The centromere-centrosome complex plays a key role in mitosis (5, 6). Centromere protein H (CENP-H) can bind to themselves, centromere protein A (CENP-A), centromere protein B (CENP-B), or centromere protein C (CENP-C) to form protein polymers localized to the centromere plate. Studies have confirmed that CENP-H is overexpressed in renal cell carcinoma (RCC), GC, hypopharyngeal squamous cell carcinoma, nasopharyngeal carcinoma, endometrial carcinoma (EC), lung cancer (LC), cervical cancer, esophageal cancer (ESCA), liver cancer, colorectal cancer (CRC), oral squamous cell carcinoma (OSCC), breast cancer (BC), and tongue cancer. CENP-H overexpression has been positively correlated to the poor prognosis, pathological stage, T stage and lymph node metastasis of patients with various cancers (7–26). Quan et al., reported that CENP-H protein and messenger ribonucleic acid (mRNA) expression levels were significantly increased in GC cells. Interference with CENP-H expression could inhibit GC cell growth, proliferation, and clonal formation by inhibiting survivin expression (8, 9). Li et al., observed that inhibition of CENP-H in lung adenocarcinoma (LUAD) cells reduced their migration, proliferation, and invasion and increased their sensitivity to cisplatin (23). The present review summarizes the roles and mechanisms of CENP-H in cancer and its clinical value. It also proposes a new candidate molecule for treating patients with cancer.

## 2 Expression levels of CENP-H in cancer

Studies have uncovered that CENP-H is overexpressed in various cancer tissues in the **Table 1** (7–22). Specifically, CENP-H is overexpressed in tissues of RCC, GC, hypopharyngeal squamous cell carcinoma, nasopharyngeal carcinoma, EC, LC, cervical cancer, ESCA, liver cancer, CRC, OSCC, BC, and tongue cancer (7–22). In addition, CENP-H is overexpressed in cells in RCC (A704, 786-O, and ACHN), GC (AGS, MGC803, BGC823, HGC27, MKN45, MKN28, and SGC7901), nasopharyngeal carcinoma (6-10B, C666, CNE1, CNE2, SUNE-1, 5-8F, and SGC7901), LC (95D, A549, H1299, hLAMP, and PAa), ESCA (108CA and KYSE140), liver cancer (Hep3B, HepG2, HHCC, SMMC7721, and MHHC97H), BC (MCF10A, MCF7, MDA231 and MDAMB435S), and tongue cancer (TSCCa and Tca8113) (7–9, 11, 13, 16–18, 21, 22). These studies collectively suggest that CENP-H is abnormally overexpressed in pan-cancer and acts as a carcinogenic factor.

**Table 1:** CENP-H expression in cancer tissues and cells.

Cancer type	Expression in tissues	N	Expression in cells	Cancer cell lines	Normal cells	Ref
RCC	up-regulation	130	up-regulation	A704, 786-O, ACHN	HK-2	(7)
GC	up-regulation	166	up-regulation	AGS, MGC803, BGC823, HGC27, MKN45, MKN28, SGC7901	GES-1	(8, 9)
HC	up-regulation	112	–	–	–	(10)
NC	up-regulation	160	up-regulation	6-10B, C666, CNE1, CNE2, SUNE-1, 5-8F, Hone1	NPEC	(11)
EC	up-regulation	57	–	–	–	(12)
LC	up-regulation	223	up-regulation	95D, A549, H1299, hLAMP, PAa	NLEC	(13)
LUAD	up-regulation	5	–	–	–	(14)
CC	up-regulation	62	–	–	–	(15)
ESCA	up-regulation	189	up-regulation	108CA, KYSE140	NE-3	(16)
Liver cancer	up-regulation	60	up-regulation	Hep3B, HepG2, HHCC, SMMC7721 MHHC97H	L02	(17, 18)
CRC	up-regulation	15	–	–	–	(19)
OSCC	up-regulation	38	–	–	–	(20)
Breast cancer	up-regulation	307	up-regulation	MCF10A, MCF7, MDA231, MDAMB435S.	BN6	(21)
Tongue cancer	up-regulation	168	up-regulation	TSCCa, Tca8113	TEC	(22)

RCC, Renal cell carcinoma; GC, Gastric cancer; HC, Hypopharyngeal cancer; NC, Nasopharyngeal carcinoma; EC, Endometrial cancer; LC, Lung cancer; LUAD, Lung adenocarcinoma; CC, Cervical cancer; ESCA, Esophageal cancer; CRC, Colorectal cancer; OSCC, Oral squamous cell carcinoma.

## 3 Roles of CENP-H in cancer

The processes of growth and metastasis are essential to cancer. Inhibiting these processes will inhibit cancer progression and improve the prognosis of patients with cancer. Inhibiting CENP-H can delay cancer growth and metastasis, and promote the sensitivity of cancer cells to cisplatin (25). CENP-H overexpression promotes cancer cell proliferation in RCC (ACHN and 786-O), GC (AGS, HGC27, and GES-1), hypopharyngeal squamous cell carcinoma (FaDu), EC (Ishikawa and HEC-1A), LC (A549 and DDP), liver cancer (Hep3B), CRC (HCT116, RKO, and LoVo), and tongue cancer (Tca8113) (7–13, 18, 19, 22–25). CENP-H was found to inhibit cell apoptosis in RCC (ACHN and 786-O) cells, hypopharyngeal squamous cell carcinoma (FaDu) cells, EC (Ishikawa and HEC-1A) cells, and liver cancer (Hep3B) cells (7, 10, 12, 18). In addition, CENP-H promoted the cell cycle transition in EC (Ishikawa and HEC-1A) cells and tumor formation in nude mice with liver cancer (Hep3B) (12, 18). CENP-H can also promote cancer cell metastasis and sensitivity to cisplatin, which in turn affects cancer progression (**Table 2**). In particular, CENP-H can promote the invasion and migration of EC (Ishikawa and HEC-1A) and LC (A549 and DDP) cells (12, 23). It can also promote cisplatin resistance in LUAD (A549 and DDP) cells and facilitate CRC cells’ resistance to radiation therapy and rapamycin (23, 25).

**Table 2:** *In vitro* functional characterization of CENP-H in cancer.

Cancer type	Expression pattern	Proliferation	Cell cycle	Apoptosis	Metastasis	Drug sensitivity	Ref
RCC	Inhibition	Inhibition	–	Promotion	–	–	(7)
GC	Overexpression	Promotion	–	–	–	–	(8, 9)
HC	Inhibition	Inhibition	–	Promotion	–	–	(10)
EC	Inhibition	Inhibition	Inhibition	Promotion	Inhibition	–	(12)
Liver cancer	Inhibition	Inhibition	–	Promotion	–	–	(18)
CRC	Overexpression	Promotion	–	–	–	Promotion	(19, 24, 25)
Tongue cancer	Inhibition	Inhibition	–	–	–	–	(22)
LC	Inhibition	Inhibition	–	–	Inhibition	Inhibition	(13, 23)

RCC, Renal cell carcinoma; GC, Gastric cancer; HC, Hypopharyngeal cancer; NC, Nasopharyngeal carcinoma; EC, Endometrial cancer; LC, Lung cancer; LUAD, Lung adenocarcinoma; CC, Cervical cancer; ESCA, Esophageal cancer; CRC, Colorectal cancer; OSCC, Oral squamous cell carcinoma.

## 4 Mechanisms associated with CENP-H involvement in cancer growth and metastasis

CENP-H can participate in cancer cell growth and metastasis through multiple signaling mechanisms. In GC and tongue cancer cells, CENP-H overexpression can increase survivin expression, thereby promoting cancer cell growth (9, 22). On the contrary, inhibiting CENP-H expression in LUAD cells can delay cell growth and metastasis by downregulating the expression of phosphorylated AKT (p-AKT), phosphorylated extracellular signal-regulated kinase (p-ERK), and phosphorylated p38 (p-p38) and promoting the sensitivity of LUAD cells to cisplatin (23). Inhibiting CENP-H expression can upregulate the expression of caspase-3 and B-cell lymphoma-2-associated X (Bax) protein and inhibit the expression of B-cell lymphoma-2 (Bcl-2) and Ki-67 proteins in liver cancer cells (18). In addition, CENP-H may be involved in the proliferation and apoptosis of liver cancer cells through the mitochondrial apoptosis pathway (18). CENP-H can promote CRC progression and modulate response to rapamycin by inhibiting the mechanistic target of rapamycin (mTOR) signaling pathway through interaction with Golgi phosphoprotein 3 (GOLPH3) (25).

CENP-H has also been observed to be regulated by carcinogenic and tumor suppressor factors (9, 12, 26). Specifically, CENP-H promotes EC cell proliferation, migration, and invasion, and it inhibits apoptosis, which can be reversed by overexpression of miR-612. Long non-coding ribonucleic acid (lncRNA) plasmacytoma variant translocation 1 (PVT1) can promote the malignant progression of EC through the miR-612/CENP-H signaling axis (12). The expression of specificity protein 1 (Sp1) and Sp3 is significantly increased in nasopharyngeal carcinoma cells. Inhibiting the activities of Sp1 or Sp3 can reduce the level of CENP-H, thus affecting the growth of nasopharyngeal carcinoma (26). In summary, CENP-H is involved in cancer growth and metastasis through PI3K/AKT, survivin, and mitochondrial apoptosis signaling mechanisms and can be regulated by lncRNA PVT1/miR-612, Sp1, or Sp3.

## 5 CENP-H overexpression and its significant association with poor prognosis in patients with cancer

Patients with cancer who exhibit overexpression of some carcinogenic factors often present a poor prognosis (**Table 3**). CENP-H overexpression has been positively associated with poor overall survival (OS) in patients with RCC, GC, nasopharyngeal cancer, EC, LC, cervical cancer, esophageal squamous cell carcinoma (ESCC), liver cancer, BC, and tongue cancer (7, 9, 11–13, 15–17, 20–22), and is significantly associated with short relapse-free survival in patients with hypopharyngeal squamous cell carcinoma (10). CENP-H overexpression has also been positively correlated with pathological stage, T stage, and lymph node metastasis of patients with cancer, and is significantly correlated with pathological stage and T stage in patients with RCC, GC, hypopharyngeal squamous cell carcinoma, nasopharyngeal carcinoma, EC, LC, cervical cancer, ESCC, liver cancer, OSCC, BC, and tongue carcinoma. It is also significantly correlated with the expression of the proliferation factor Ki-67 in patients with GC, LC, OSCC, and BC (7, 9–13, 15–17, 20–22). These results suggest that CENP-H overexpression helps predict poor prognosis in patients with such cancers.

**Table 3:** CENP-H expression is significantly correlated with the prognosis of patients with cancer.

Cancer type	Prognostic indicator	Associated clinical features	Ref
RCC	OS	Distant metastasis, clinical stage, Fuhrman grade	(7)
GC	OS	Tumor size, T stage, pathological stage, lymph node metastasis, Ki-67	(9)
HC	RFS	Pathological stage, alcohol consumption	(10)
NC	OS	T stage, pathological stage	(11)
EC	OS	pathological stage	(12)
LC	OS	T stage, pathological stage, Ki-67	(13)
CC	OS	pathological stage	(15)
ESCA	OS	T stage, pathological stage, Gender	(16)
Liver cancer	OS	Tumor size, grade, pathological stage	(17)
OSCC	–	pathological stage, Ki-67	(20)
Breast cancer	OS	Pathological stage, Ki-67, T stage, lymph node metastasis	(21)
Tongue cancer	OS	Pathological stage, T stage	(22)

RCC, Renal cell carcinoma; GC, Gastric cancer; HC, Hypopharyngeal cancer; NC, Nasopharyngeal carcinoma; EC, Endometrial cancer; LC, Lung cancer; LUAD, Lung adenocarcinoma; CC, Cervical cancer; ESCA, Esophageal cancer; CRC, Colorectal cancer; OSCC, Oral squamous cell carcinoma; RFS, relapse-free survival.

## 6 Conclusion

CENP-H plays a role in organizing and stabilizing centromeres during cell mitosis. CENP-H is overexpressed in RCC, GC, hypopharyngeal squamous cell carcinoma, nasopharyngeal carcinoma, EC, LC, cervical cancer, ESCA, liver cancer, CRC, OSCC, BC, and tongue cancer. This overexpression is correlated with the prognosis, pathological stage, T stage, and lymph node metastasis of patients with such cancers. CENP-H can promote cancer growth and metastasis through PI3K/AKT, Survivin, and mitochondrial apoptosis signaling mechanisms, and it can be regulated by lncRNA PVT1/miR-612, Sp1, or Sp3. Presently, studies on CENP-H are not comprehensive in cancer. First, the interaction between CENP-H and other proteins can be extensively researched to uncover its intrinsic mechanism in regulating cell division. Second, the roles and signaling pathways of CENP-H in normal cells is still unknown, which is also worth exploring in the future. Finally, the structural and functional information of CENP-H can be utilized to develop new drugs to treat cancer. Overall, further studies of CENP-H will contribute to a deeper understanding of the mechanisms of cell division regulation and may provide new candidate molecules and therapeutic targets for disease treatment.
